# Impact of engaging security personnel on access and polio immunization outcomes in security-inaccessible areas in Borno state, Nigeria

**DOI:** 10.1186/s12889-018-6188-9

**Published:** 2018-12-13

**Authors:** Loveday Nkwogu, Faisal Shuaib, Fiona Braka, Pascal Mkanda, Richard Banda, Charles Korir, Samuel Bawa, Sule Mele, Mahmud Saidu, Hyelni Mshelia, Aliyu Shettima, Sisay G. Tegegne, Yared G. Yehualashet, Usman Adamu, Peter Nsubuga, Rui G. Vaz, Alemu Wondimagegnehu

**Affiliations:** 1 World Health Organization Country Representative’s Office, Abuja, Nigeria; 2 National Primary Health Care Development Agency, Abuja, Nigeria; 3 Borno State Primary Health Care Development Agency, Maiduguri, Nigeria; 4 Global Public Health Care Solutions, Atlanta, GA USA; 5 World Health Organization, Geneva, Switzerland

**Keywords:** Security inaccessible areas, Civilian joint task force, Supplemental immunization activities, Lot quality assurance sampling, Outbreak response, Missed children, Zero-dose children

## Abstract

**Background:**

Nigeria was polio free for almost 2 years but, with the recent liberation of areas under the captivity of insurgents, there has been a resurgence of polio cases. For several years, these inaccessible areas did not have access to vaccination due to activities of Bokoharam, resulting in a concentration of a cohort of unvaccinated children that served as a polio sanctuary. This article describes the processes of engagement of security personnel to access security-compromised areas and the impact on immunization outcomes.

**Methods:**

We assessed routine program data from January 2016 to July 2016 in security-inaccessible areas and we evaluated the effectiveness of engaging security personnel to improve access to settlements in security-compromised Local Government Areas (LGAs) of Borno state. We thereafter evaluated the effects of this engagement on postcampaign evaluation indicators.

**Results:**

From 15 LGAs accessible to vaccination teams in January 2016, there was a 47% increase in July 2016. The number of wards increased from 131 in January to 162 in July 2016, while the settlement numbers increased from 6050 in January to 6548 in July 2016. The average percentage of missed children decreased from 8% in January to 3% in July 2016, while the number of LGAs with ≥ 80% coverage increased from 85% in January to 100% in July 2016.

**Conclusion:**

The engagement of security personnel in immunization activities led to an improved access and improvement in postcampaign evaluation indicators in security-compromised areas of a Nigerian state. This approach promises to be an impactful innovation in reaching settlements in security-compromised areas.

## Background

Following the global launch to eradicate polio by 2000 [1], tremendous progress has been made with the disease now being contained all over the world except for Nigeria, Afghanistan, and Pakistan [2]. Nigeria was polio free from August 2014 to June 2016, when new polio cases were isolated in Borno state in the northeastern part of the country; this negatively impacted the global polio eradication effort as indigenous wild poliovirus (WPV) transmission now persists in three countries [3]. These countries have a high infrastructural health deficit, a high prevalence of enteric diseases, a high level of malnutrition, and a suboptimal implementation of routine immunization activities. They rely on several supplemental immunization activities (SIAs) to increase population immunity [4, 5].

The combined use of oral polio vaccine (OPV) and inactivated polio vaccine (IPV) in vaccination campaigns confers better population immunity and needs to be employed routinely in endemic countries and countries that are prone to the reintroduction of WPV [6]. The monovalent and bivalent virus strains induce higher levels of immunity than the trivalent vaccine; however, they can only elicit a response for the specific type of virus. This means that monovalent OPV1, OPV2, and OPV3 can only elicit a response for WPV1, WPV2, and WPV3, respectively [4].

Some oral polio vaccine-derived viruses may be genetically altered during replication on excretion, which could then circulate through and cause paralysis in populations with low immunity; these viruses are called circulating vaccine-derived polioviruses (cVDPVs), and there is surveillance in place to detect and test specimens in World Health Organization-accredited laboratories [7, 8]. There are standardized global polio eradication initiative performance indicators that are used to evaluate the quality of acute flaccid paralysis (AFP) surveillance and to identify where WPV transmission might go undetected [9–12]. To interrupt the cVDPV2 that was largely responsible for the cVDPV cases, the strategic group of experts (SAGE) proposed the withdrawal of type 2 OPV by globally switching from trivalent OPV (tOPV) to bivalent OPV (bOPV) (types 1 and 3) in April 2016 [3]. The increased risk for outbreaks of cVDPV2 necessitated a strategic path to eventual cessation of OPV and the polio eradication end-game strategic plan of 2013–2018, which provided comprehensive, long-term strategies to deliver a polio-free world by 2018 [13, 14].

Borno state, in the northeast of Nigeria, recorded a cVDPV2 from an environmental sample taken on 23 May 2013 from the Abbaganaram site in the Lamisula ward of Maiduguri Metropolitan Council (MMC). The virus was an orphan virus, although it was closely related to a virus isolated in an adjacent community in Konduga, Nigeria, in 2003 and Chad in 2012. The laboratory result came after the switch from tOPV to bOPV. The burden was then on the state to conduct three outbreak responses while ensuring that there was an effective vaccine management system in place that could account for all the vials of monovalent OPV (mOPV) type 2. There was also the challenge of insecurity from an insurgency that rendered about 50% of the state inaccessible. The need to ensure that vaccinations got to most of these areas was strengthened by the fact that there was a plan to withdraw mOPV2 after the outbreak response.

In an attempt to reach these security-inaccessible areas, the government and the natives adopted several innovative ways to leverage existing security structures to deliver immunization services to these underserved inaccessible populations with low immunity. Security personnel were engaged to support the vaccination teams to get access to these inaccessible areas.

This article describes the processes of engagement of security personnel in the three outbreak response campaigns in Borno state and highlights the impact on immunization outcomes between May 2016 and July 2016. The goal of this article is to document and share a feasible approach for accessing conflict areas in Africa.

## Methods

### Description of the organizational structure of the state and security personnel engaged in the delivery of immunization services in Borno state

#### Civilian joint task force

The civilian joint task force (CJTF) is a community-initiated security network that is supporting the Nigerian military in the war against insurgents in Borno state. They have a President at the state level as the head, and a Chairman at the Local Government Area (LGA) level. The LGAs are further subdivided into sectors headed by commanders. The CJTF is made up of energetic young men who have organized themselves to positively apply their energy to the defense of their communities.

The CJTF are natives who may not have had any formal security training, but they are familiar enough with the environment to identify insurgents and other foreign elements that have the potential for perpetuating violence, who are then arrested by the CJTF through their command structure and subsequently handed over to the Nigerian military.

#### The Nigerian military

The Nigerian military are trained and serving members of the Nigerian Armed Forces under the 7th Mechanized Division in Borno state.

#### State emergency operation Centre (SEOC)

The SEOC is the highest decision-making body on the polio eradication initiative in Borno state. It was structured after the National Emergency Operation Center (NEOC) at the federal level. The SEOC membership consists of high-level government officers and partners who are involved in polio eradication.

### Process of engagement of the CJTF and operations

During the outbreak response to the cVPDV from an environmental sample taken on March 23 2016, we discovered that MMC, Jere LGA, and some wards in Mafa and Konduga LGAs (all in Borno state) had many eligible children outside households that could be reached using directly observed polio vaccination (DOPV), but the challenge was dealing with the huge crowds associated with DOPV in an environment that was prone to attacks by insurgents.

#### Advocacy

We developed PowerPoint presentations highlighting the benefits of DOPV as a key advocacy material and outlining a strategy for reaching the underserved. This was presented to the SEOC to solicit their agreement to the strategy. The major challenge was conducting DOPV with the attendant crowd in an unstable security environment that had witnessed several bombings with improvised explosive devices. The authors and the SEOC reasoned that engaging the CJTF could be central for a successful implementation of DOPV. The advocacy kit which emphasized the benefits and dangers of reaching the isolated populations led to a joint decision to obtain CJTF security cover for the implementation of a DOPV strategy on 13 May 2016. A strategy was drafted which needed to be acceptable to the CJTF. To facilitate their acceptance, we used the influence of the state government to market the draft strategy to the CJTF. We also studied the operational processes and effectiveness of the CJTF and, given this understanding, the team worked out a feasible strategy.

The major output of the meeting with the management of CJTF was to obtain their consent to engage their members, identify and source for the required number of CJTF members, and agree on the date for their orientation.

#### Orientation and deployment of the CJTF

We organized an orientation for the CJTF participants on their possible roles in the DOPV approach for security-compromised areas of the state. The orientation provided guidance for CJTF members to report at designated take-off points by 7 am, provide security for vaccination teams, support the teams in crowd control, report truancy of team members to senior supervisors, and give regular feedback at the ward-level review meeting.

We deployed CJTF members to work in areas they were more familiar with and where they had some level of authority. A day was used to conduct dress rehearsals of the activities expected from the CJTF. A member of the CJTF was attached to a DOPV team in their domain and they worked from the beginning to the end with their respective team members for the 2 days during which DOPV was conducted. Their commanders were supported to be able to move around and monitor all activities to ensure they were in tandem with the overall strategy. A total of 611 persons were engaged in the June 2016 outbreak response, while 874 persons were engaged in the July 2016 outbreak response.

We also engaged some CJTF members to conduct ‘hit-and-run’ vaccinations, which are discreetly conducted, rapid-response vaccination activities to boost immunity of the children with a reduced risk exposure for the field staff. This strategy involves conducting the vaccinations at such a time when the risk of insurgency is considered minimal based on intelligence reports in very specific areas. The ‘hit and run’ strategy was conducted in July 2016 SIAs in 33 settlements in five wards that were inaccessible in the Jere LGA of Borno state.

### Process of engagement of the Nigerian military

We conducted advocacy visits to the top military hierarchy in Borno state to obtain their support. The outcome of the meetings was the designation of their logistic focal points to coordinate the vaccination team’s movements. This included the agreement on routes and the level of security required and type of military hardware to be deployed. For example, whereas the teams to Dambao LGA followed the regular military escort, a special highly fortified military escort was deployed to escort the teams to Bama, Gwoza, Dikwa, Ngala, Kala-balge, Monguno, and Kukawa LGAs that were considered higher risk areas or more inaccessible LGAs.

Each team was provided with basic requirements such as vaccines for routine immunization and SIA, adequate frozen ice-packs, and donkeys or bicycles as the key vehicular movement. Automobile use was not allowed in all cases. The basic resources available to the teams were such that they would last for 5–7 days which was the agreed maximum period they were expected to stay in any of these LGAs.

As a precautionary security measure, information on the exact date and time of the movement was controlled by the military personnel. At the point of departure, there was pre-inspection of all vehicles and the ones that did not meet the required standard were not allowed to embark on the journey. The convoy was arranged in such a way that there was an armored tank in front and at the back of the convoy. In addition, all phone networks were shut down as a precautionary security measure.

We collected and transmitted data daily through text messages in areas with a cellular phone network, while areas with no network were collated when the teams returned.

The engagement with the military created a delivery corridor for the movement of vaccines, sachets of milk, detergents, and sugar to the state as they used the military network to ensure vehicles conveying these commodities were allowed to move during the curfew period and when there was a ban on vehicular movement.

As a sequel to the security-enhanced vaccination activities in these high-risk areas, we evaluated the impact of our reach using qualitative and quantitative methods. The data were taken from the direct output of the vaccination process, including the number of LGAs, wards, and settlements reached, and the number of children vaccinated by engaging security personnel. We also used security accessibility data reports, supervision checklists, daily review meeting data, outcome indicators from postcampaign evaluation data, lot quality assurance sampling (LQAS) data, and end-process monitoring data. The data were obtained from the Borno SEOC, while the postcampaign evaluation data were from the World Health Organization. We analyzed the quantitative data using Microsoft Excel.

## Results

**Fig. 1:**
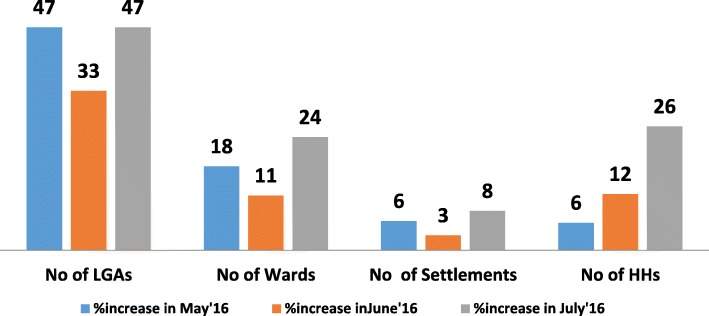
Percentage increase in Local Government Areas (LGAs), wards, settlements and households (HHs) reached by vaccination teams after the engagement of security personnel in supplemental immunization activities

The percentage of LGAs accessed increased by 47% between May 2016 and July 2016 (Fig. 1). There was also an increase in the number of wards and settlements accessed in May 2016 and July 2016. However, there was a decrease in the number of LGAs, wards, and settlements accessed in June 2016. Despite the dip in June 2016, there was still a stepwise increase in the number of households accessed in May, June, and July 2016 (Fig. 1).

**Fig. 2:**
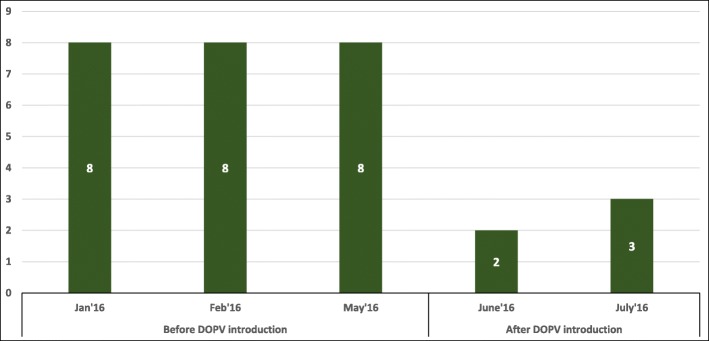
Average percentage of missed children before and after directly observed polio vaccination (DOPV) was introduced from January 2016 to July 2016

The average percentage of missed children decreased in June 2016 and July 2016 when the DOPV was conducted. The average percentage of missed children was 8% in January 2016 while it was reduced to 2 and 3% in June 2016 and July 2016, respectively, after the introduction of DOPV (Fig. 2).

Table 1 shows the percentage coverage with OPV2 in the four LGAs that implemented DOPV in Borno state. The data from the campaigns from January to July 2016 revealed that the percentage coverage increased after the introduction of DOPV in June 2016. The highest coverage with OPV before the introduction of DOPV was 123% in May 2016 in Jere LGA. After the introduction of DOPV, the highest coverage was 154% in Jere LGA.

**Table 1:** Percentage coverage in LGAs before and after directly observed polio vaccination in Jere, Konduga, Mafa, and MMC in January to July 2016

LGA	No. of wards	% coverage in January	% coverage in February	% coverage in May	% coverage in June	% coverage in July
Jere	12	114	118	123	139	154
Konduga	1	96	100	99	106	123
Mafa	1	110	113	117	123	139
MMC	15	99	100	102	107	125

*LGA* Local Government Area, *MMC* Maiduguri Metropolitan Council

**Fig. 3:**
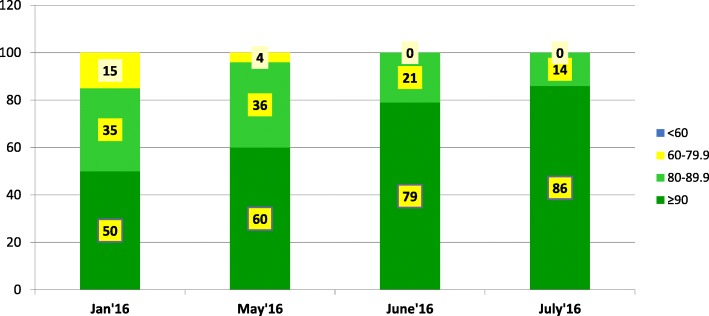
Lot quality assurance sampling survey results in Borno state in the May to July 2016 outbreak response

LQAS survey data revealed that, in January 2016, 85% of the LGAs had coverage ≥ 80% while, with the engagement of security personnel, all the LGAs had over 80% coverage in the July 2016 campaign (Fig. 3).

The ‘hit and run’ strategy was conducted with the support of the CJTF to reach 33 inaccessible settlements in five wards where a total of 10,074 eligible children were vaccinated (Table 2). This was realistically the only way of reaching such populations that otherwise would have been missed.

**Table 2:** Inaccessible settlements reached using the ‘hit and run’ strategy of July 2016 in five wards in Jere Local Government Area, Borno state

Ward	No. of settlements	No. of children vaccinated
Tuba	10	433
Galtimari	7	8780
Gungulong	9	578
Alau	2	132
Old Maiduguri	5	151
Total	33	10,074

Table 3 reveals an increase in access to inaccessible settlements from 231 in May 2016 to 359 in July 2016, while the number of households visited increased from 21,240 to 42,354. It was also shown that a total of eight cases of AFP were detected in the three campaigns.

**Table 3:** Vaccination data report of inaccessible settlements that were accessed and AFPs detected with support of the military in the May 2016, June 2016, and July 2016 campaign in Borno state

	No. of LGAs	No. of wards	No. of settlements	No. of households visited	Total no. vaccinated with OPV	No. of AFPs
May 2016	7	17	231	21,240	172,331	4
June 2016	4	11	248	32,107	134,805	1
July 2016	8	18	359	42,354	204,225	3

*AFP* acute flaccid paralysis, *LGA* Local Government Area, *OPV* oral polio vaccine

**Fig. 4:**
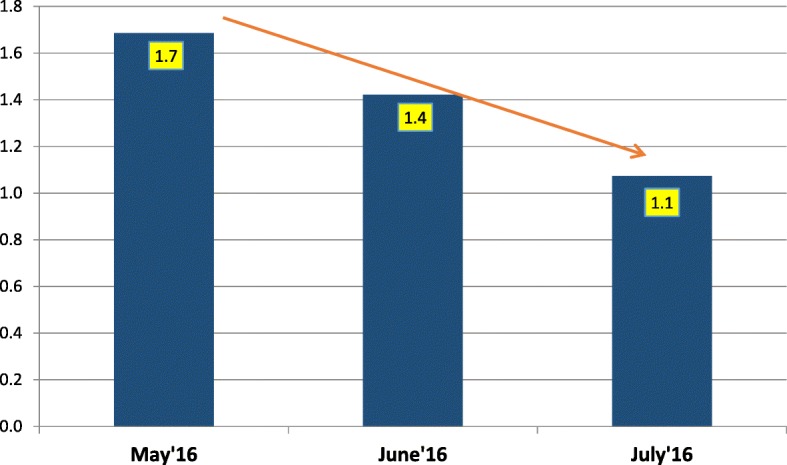
Trend of percentage zero-dose children in inaccessible LGAs vaccinated with a military escort

Data from the tally sheet showed that the trend in percentage of zero-dose children (i.e., children who had never received a dose of OPV) declined between May and July 2016. In May 2016, the zero-dose percentage was 1.7%, in June 2016 it was 1.4%, while in July 2016 it was 1.1% (Fig. 4).

## Discussion

Our engagement of security personnel in the polio SIA in Borno state led to an increase in the number of LGAs, wards, settlements, and households accessed by the polio vaccination teams. The systematic engagement of security personnel improved access to security-compromised settlements. This is similar to the findings of Ager et al. in 2015 in Yobe State, where counter-insurgency arrangements were made with the joint task force leading to security personnel escorting patients from their homes to health facilities when contacted through a medical emergency telephone number [15]. Our engagement with security personnel allowed free movement of teams and logistics during curfew periods, unlike with Ager et al. 2005, where imposition of a curfew led to travel restrictions and limited access to security-compromised settlements [15], thus expanding the service delivery window.

We also found that the number of children vaccinated with the engagement of the security personnel increased. Similarly, the number vaccinated in the four LGAs that conducted DOPV with support from members of the CJTF increased. These are similar to the findings of Fekadu et al. in Angola [16], where it was possible to engage the military to reach children in insecure areas with vaccines and other interventions. Similar findings from Dadgar et al. [17] also revealed high measles vaccination coverage with the support of the military in Afghanistan despite the security challenges.

In our specific context, we can confirm that strategic engagement and deployment of security personnel and local intelligence can help AFP detection and investigation in security-compromised and conflicted areas. This has improved the number of times polio teams interface with the community members, similar to the findings of Fekadu et al. [16] where the partnership with the military in Angola improved the AFP detection and notification and other epidemic-prone diseases in areas with limited access to health services.

The implementation of the polio SIAs with the support of security personnel has improved postimplementation evaluation outcomes in Borno state. The analyses of LQAS surveys conducted revealed an increase in the number of LGAs accepted at ≥ 90% coverage. Similarly, the end-process monitoring outcome revealed a decrease in the percentage of missed children after we engaged security personnel to conduct DOPV. These security personnel guaranteed unhindered access to many settlements in the LGAs that were not previously accessed because of insecurity.

We also discovered that the percentage zero-dose was decreased, which is an indication that the cohorts who were previously not reached because of the insurgency are gradually being reached, and this is expected to improve the herd immunity that will interrupt transmission of poliovirus.

There were some limitations with the study. These include the fact that many other interventions were concurrently happening with the engagement of security personnel, and hence the improved outcome seen may also be attributed to some of these other interventions, thus making it difficult to statistically prove a positive outcome of engaging security personnel. However, the fact that these other interventions were ongoing before the engagement of the security personnel but the outcome did not show the improvement seen makes it easier to appreciate the contributions of the security personnel to the improved outcome. Another limitation was the high population dynamics in the state. As the war against insurgents was ongoing, there was a resultant movement of people in search of areas with relative peace.

Despite these limitations, the engagement of security personnel played a critical role in the improvement in the number of LGAs, wards, settlements, and households accessed by the polio vaccination team. This improved access invariably led to increased coverage and reduction in the number of missed children and overall improvement in outcome.

## Conclusion

In conclusion, the engagement of security personnel to accompany polio SIA teams was a watershed moment in the quality of polio SIAs in Borno state. There is a need to sustain this approach as it is one feasible way to ensure the safety of teams while serving as a way of improving access to polio vaccines in security-compromised areas of Borno state. We recommend that the lessons learned from this approach should be applied in improving routine immunization in security-compromised areas in conflict-prone areas of Africa.

## Abbreviations

AFP: Acute flaccid paralysis
bOPV: Bivalent oral polio vaccine
CJTF: Civilian joint task force
cVPDV: Circulating vaccine-derived poliovirus
DOPV: Directly observed polio vaccination
IPV: Inactivated polio vaccine
LGA: Local Government Area
LQAS: Lot Quality Assurance Sampling
MMC: Maiduguri Metropolitan Council
mOPV: Monovalent oral polio vaccine
NEOC: National Emergency Operation Center
OPV: Oral polio vaccine
SAGE: Strategic group of experts
SEOC: State Emergency Operation Center
SIA: Supplemental immunization activity
tOPV: Trivalent oral polio vaccine
WPV: Wild poliovirus
